# Genomic and Pathological Characterization of Multiple Renal Cell Carcinoma Regions in Patient With Tuberous Sclerosis Complex: A Case Report

**DOI:** 10.3389/fonc.2021.691996

**Published:** 2021-05-12

**Authors:** Tetsuya Yamamoto, Taigo Kato, Koji Hatano, Atsunari Kawashima, Takeshi Ujike, Shinichiro Fukuhara, Hiroshi Kiuchi, Ryoichi Imamura, Naokazu Ibuki, Kazuma Kiyotani, Masako Kurashige, Eichi Morii, Kazutoshi Fujita, Norio Nonomura, Motohide Uemura

**Affiliations:** ^1^ Department of Urology, Osaka University Graduate School of Medicine, Osaka, Japan; ^2^ Department of Urology, Osaka Medical College, Osaka, Japan; ^3^ Cancer Precision Medicine Center, Japanese Foundation for Cancer Research, Tokyo, Japan; ^4^ Department of Pathology, Osaka University Graduate School of Medicine, Osaka, Japan

**Keywords:** tuberous sclerosis complex, renal cell carcinoma, papillary renal cell carcinoma, whole-exome sequencing, cancer gene

## Abstract

Tuberous sclerosis complex is a genetic disorder characterized by facial angiofibromas, intellectual disability, epilepsy, and tumor formation in multiple organs, including the kidney. Renal cell carcinoma occurs in 2%–4% of patients with tuberous sclerosis complex, often developing multiply and bilaterally. Renal cell carcinoma associated with this genetic disorder may include complex tumor heterogeneity caused by the spatially different mutational landscape. Herein, we report the case of a female patient with tuberous sclerosis complex who developed multiple renal tumors. A 44-year-old female patient with tuberous sclerosis complex developed three different histological types of tumor—angiomyolipoma, clear cell renal cell carcinoma, and papillary renal cell carcinoma—in the left kidney at first renal cell carcinoma recurrence. The papillary renal cell carcinoma was morphologically atypical, indicating that its occurrence was associated with the genetic disorder. Furthermore, whole-exome sequencing revealed distinct patterns of somatic mutation in the three tumor types, and the atypical papillary renal cell carcinoma possessed a different mutational landscape than that of typical papillary renal cell carcinomas. Our findings indicate that tumors associated with tuberous sclerosis complex may be diagnosed with careful pathological examination. Furthermore, somatic mutation profiles of these tumors revealed their unique features, providing important information for further understanding the mechanism of multiple tumor development in patients with tuberous sclerosis complex.

## Introduction

Tuberous sclerosis complex (TSC) is a rare autosomal dominant genetic disorder with manifestations such as facial angiofibromas, intellectual disability, and epilepsy occurring in 1 of every 6,000 births (1–3). This disorder is associated with mutations in *TSC1* or *TSC2*; these genes encode proteins (hamartin and tuberin) that act as a complex involved in tumor suppression and regulation of the rapamycin (mTOR) signaling pathway mammalian target.

Disorders affecting the mTOR pathway comprise clinical features indicating a predisposition to tumor development in multiple organs, including the kidney. Specifically, renal tumors are found in 70%–80% of patients with TSC (4). The three major types of renal manifestations occurring in these patients are angiomyolipoma (AML), renal cyst, and renal cell carcinoma (RCC). TSC-associated RCC occurs in 2%–4% of patients with TSC (5), an estimated incidence rate higher than that in the general population. Moreover, TSC-associated RCC often occurs in the younger individuals, requiring close monitoring for recurrent RCC throughout their lifetime (5, 6). TSC-associated RCC is also characterized by multiple occurrences in the same patient (7, 8). This renal tumor occurs bilaterally in approximately 30% of cases and often comprises several types of morphology, including clear cell, papillary, and chromophobe RCC, as well as benign AML (5, 7, 9).

Herein, we describe a case of a patient with TSC who presented with three types of tumors—clear cell RCC, papillary RCC, and AML—in the same kidney. In the present study, we demonstrated that immunohistochemical analysis is an important tool to identify the occurrence of RCC associated with TSC, especially when the patient was not previously diagnosed with this genetic disorder. Moreover, we examined the somatic mutation profiles of the tumors, highlighting their unique features and mutational landscapes, which may contribute to understanding the mechanism involved in multiple tumor formation in patients with TSC.

## Case Presentation

**Figure 1:**
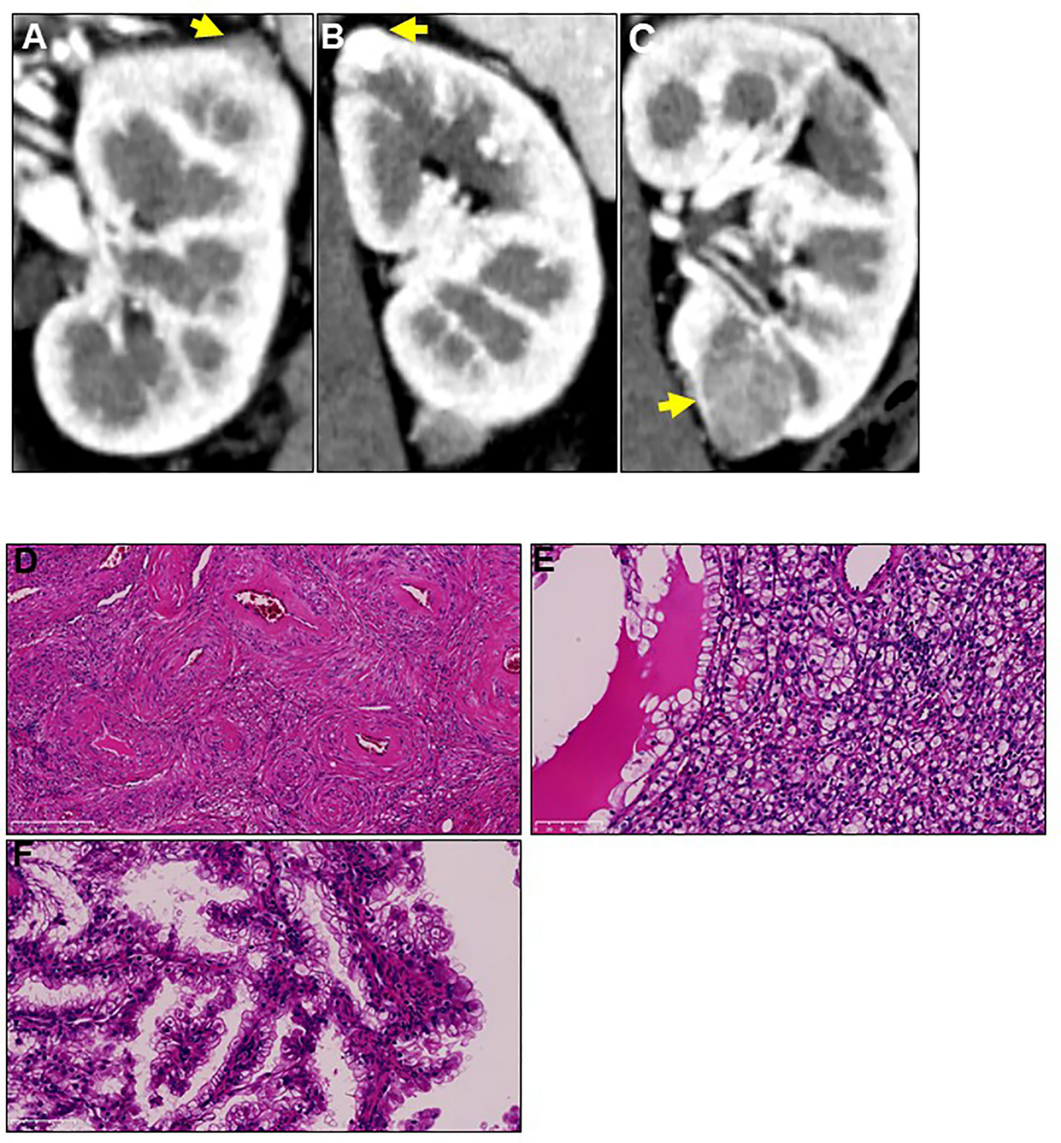
Three different tumors located in the patient’s left kidney at first recurrence. Two years after radical right-kidney nephrectomy, the patient was diagnosed with three different tumors **(A-C)** in her left kidney on computed tomography examination. Yellow arrows show the three tumors. The patient underwent left-kidney partial nephrectomy for all tumors, and immunohistochemical analysis showed that their histopathological types were **(D)** angiomyolipoma, **(E)** clear cell renal cell carcinoma, and **(F)** papillary renal cell carcinoma. Magnification: 200× for hematoxylin and eosin staining.

A 44-year-old Japanese woman was referred to our hospital for treatment of a recurrent tumor in the left kidney. Five years prior to this referral, the patient underwent right-kidney nephrectomy for RCC and received a histopathological diagnosis of clear cell RCC (pT1aN0M0) at another institution. Two years after this, computed tomography (CT) imaging identified three tumors in her left kidney; the patient underwent left-kidney partial nephrectomy for these tumors (**Figures 1A–C**). Histopathological examination determined that the tumors were AML, clear cell RCC (pT1a), and papillary RCC (pT1a) (**Figures 1D–F**). A periodic CT examination 3.5 years later revealed the tumor recurrence in her left kidney.

**Figure 2:**
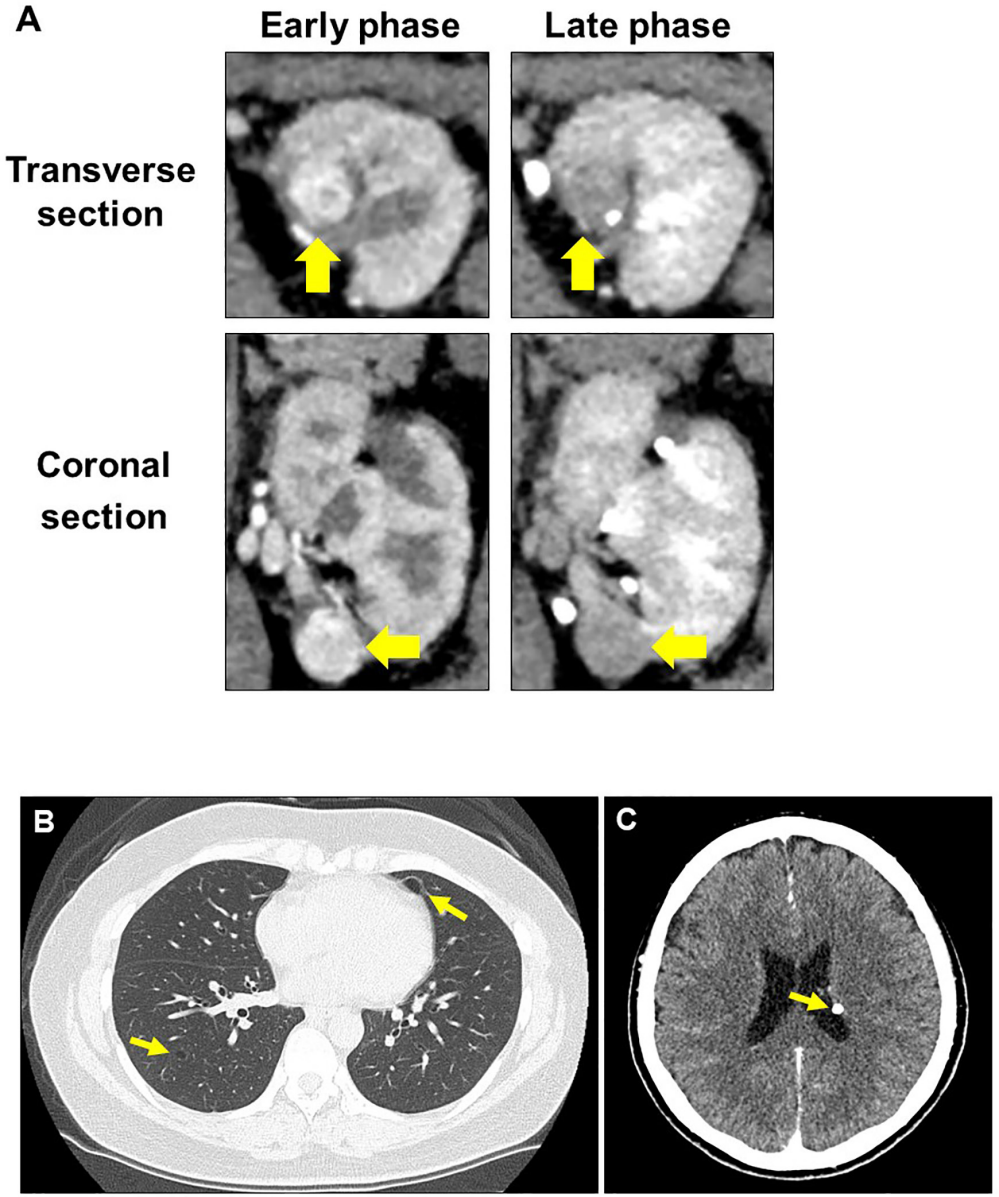
Radiographic evaluation at second recurrence. **(A)** Computed tomography examination shows typical findings of clear cell renal cell carcinoma in the left kidney. **(B)** Lung lymphangioleiomyomatosis. **(C)** Subependymal nodule at the left lateral ventricular wall of the brain.

Upon initial visit to the Osaka University Hospital, abdominal CT scan showed a renal mass (diameter: 22 mm) with early enhancement in the left kidney (**Figure 2A**). Additional screening tests revealed the presence of lung cysts and calcifications in the left ventricular wall of the brain (**Figures 2B, C**), leading to the suspicion of TSC. Moreover, physical examination revealed five major (ungual fibromas, shagreen patches, lymphangioleiomyomatosis, subependymal nodule, and angiomyolipoma) and one minor (dental enamel pits) TSC manifestations according to clinical and genetic diagnostic criteria (10). Combining these findings, we diagnosed the patient with recurrence of left-kidney RCC and TSC.

Considering the high recurrence rate of TSC-associated RCC, the patient received CT-guided percutaneous cryoablation for the left-kidney recurrent tumor to maintain maximal renal function. Tumor biopsy performed after cryoablation identified the tumor as clear cell RCC by immunohistochemical staining. To evaluate kidney function, we calculated the estimated glomerular filtration rate (eGFR) before and 3 mo after cryoablation. The rate of kidney functional deterioration was 3.5%. The patient remained recurrence-free for 3 years without renal function deterioration.

### Histopathological Features of Renal Cell Carcinoma

**Figure 3:**
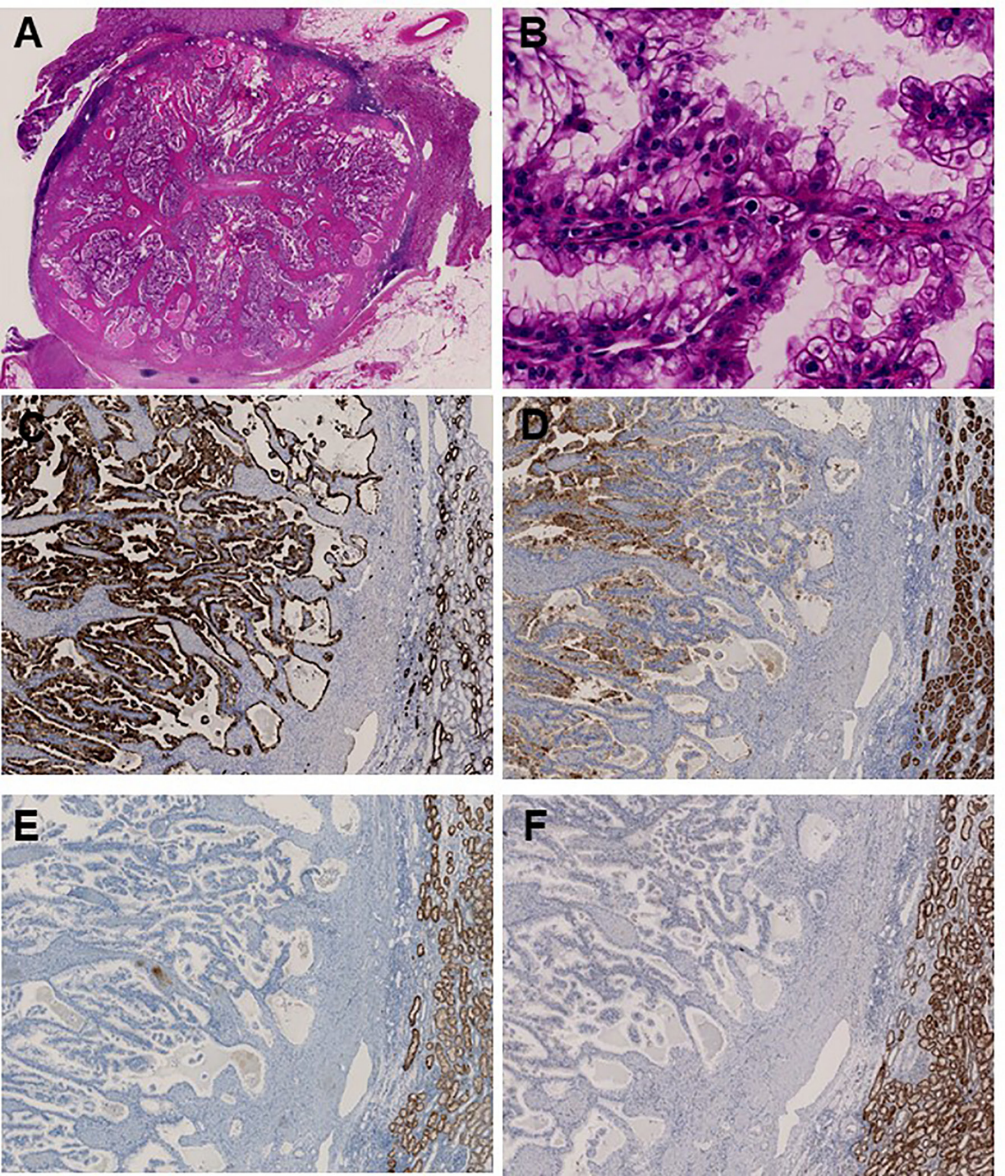
Immunohistochemical analysis identifies papillary renal cell carcinoma associated with tuberous sclerosis complex. **(A)** Main tumor nodule surrounded by thick fibrous stroma on low power; **(B)** Prominent papillary architecture lined by large clear cells with delicate eosinophilic cytoplasmic thread-like strands, which occasionally appeared more outstanding and aggregated to form eosinophilic globules on high power. Immunohistochemical analysis revealed positive staining for **(C)** CK7 and **(D)** CD10, whereas **(E)** SDHB and **(F)** AMACR were negative.

Upon the diagnosis of a second RCC recurrence, we retrospectively examined the three tumors that were identified at first recurrence considering that TSC-associated RCC has several unique features. We observed prominent papillary architecture lined by clear cells with delicate eosinophilic cytoplasmic thread-like strands that occasionally appeared more prominent and aggregated to form eosinophilic globules in the papillary RCC sample (**Figures 3A, B**). Immunohistochemical analysis revealed that CK7 and CD10 were positive, whereas succinate dehydrogenase subunit B (SDHB) and α-methylacyl-CoA racemase (AMACR) were negative (**Figures 3C–F**). These findings demonstrated that the characteristics of the papillary RCC in our patient were consistent with those of TSC-associated papillary RCC, which was recently reported as a new type of papillary tumor occurring in patients with TSC (11).

### Somatic Mutations and Alterations in Cancer-Related Genes

**Figure 4:**
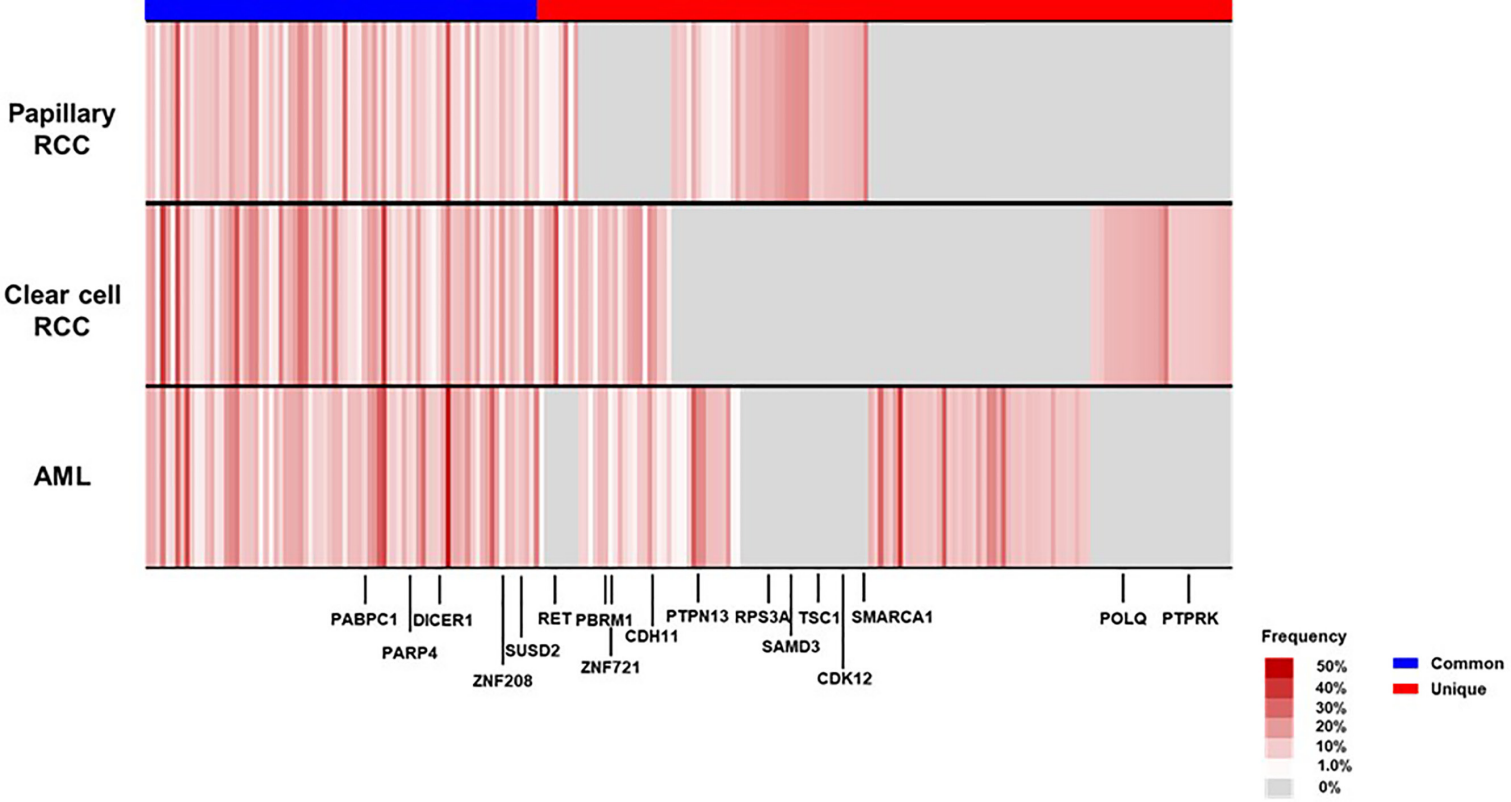
Mutational landscape of the three renal tumors in the patient’s left kidney at first recurrence. We visualized the somatic mutation profiles of each tumor—angiomyolipoma (AML), clear cell renal cell carcinoma (RCC), and papillary RCC—as heat maps (black-colored genes indicate driver gene mutations in many cancer types). *CDH11*, Cadherin 11; *CDK12*, Cyclin Dependent Kinase 12; *DICER1*, Dicer 1; *PABPC1*, Poly(A) Binding Protein Cytoplasmic 1; *PARP4*, Poly(ADP-Ribose) Polymerase Family Member 4; *PBRM1*, Polybromo 1; *POLQ*, DNA Polymerase Theta; *PTPN13*, Protein Tyrosine Phosphatase Non-Receptor Type 13; *PTPRK*, Protein Tyrosine Phosphatase Receptor Type K; *RET*, Ret Proto-Oncogene; *RPS3A*, Ribosomal Protein S3A; *SAMD*3, Sterile Alpha Motif Domain Containing 3; *SMARCA1*, SWI/SNF Related, Matrix Associated, Actin Dependent Regulator Of Chromatin, Subfamily A, Member 1; *SUSD2*, Sushi Domain Containing 2; *TSC1*, TSC Complex Subunit 1; *ZNF208*, Zinc Finger Protein 208; *ZNF721*, Zinc Finger Protein 721.

To characterize the intra-tumoral genetic heterogeneity of this case, we performed whole-exome sequencing using genomic DNA extracted from the tumors surgically resected at first recurrence. We obtained an average sequencing depth of 82.3× per base and identified 221 non-silent mutations and insertions/deletions (indels) (124–154 non-silent mutations per tumor, **Additional Table 1**). We found that 36.7% of these somatic mutations—including cancer driver genes such as *PABPC1* and *DICER1*, which are common in parental clones of many cancer types—were shared among the three tumors (common mutations, **Figure 4**). Some mutations were uniquely observed in one or two tumors (unique mutations), which may have been acquired during individual tumor formation, contributing to the high intra-tumoral genetic heterogeneity.

Interestingly, in our patient’s papillary RCC sample, 37.1% of common mutations and 25.5% of unique mutations were not previously reported as non-silent mutations in the Cancer Genome Atlas database (**Additional Figure 1**). Regarding *TSC1* and *TSC2* mutations, TSC-associated papillary RCC harbored frameshift *TSC1* mutation (c.2142del, p.Asn715fs), a pathogenic variant for patients with TSC reported in the ClinVar database. Conversely, *TSC1* and *TSC2* germline mutations were not found in our patient, implying that she may possess the phenotype with mosaic forms of TSC.

## Discussion

The occurrence of RCC in patients with TSC has been recognized for several decades. Unlike typical RCC, TSC-associated RCC has several unique features, including early onset (around 40 years old), predominance in female patients, and multiple and bilateral tumors with distinct pathological characteristics (1, 2, 5, 8). Therefore, because chronic kidney disease is a common cause of death in patients with TSC, physicians need to carefully determine therapeutic strategies for TSC-associated RCC to avoid renal function impairment (4). Herein, we described a case of TSC-associated RCC and identified distinct patterns of pathological findings and mutational landscapes among clear cell RCC, papillary RCC, and AML occurring in the same kidney, leading to several important implication**s**.

First, upon immunohistochemical analysis, we identified several TSC-associated papillary RCC characteristics that differed from typical papillary RCC, including prominent papillary architecture, abundant clear cell cytoplasm, uniformly deficient SDHB expression, and negative staining for AMACR (11). These findings strongly indicate the presence of TSC, especially in patients displaying fewer clinical features associated with this disorder. Considering that TSC-associated RCC may show multiple and bilateral recurrence, the timely recognition of this atypical form of RCC using immunohistochemical analysis may allow treatment with local therapy instead of radical nephrectomy, possibly avoiding the development of chronic kidney disease in these patients.

Second, we identified that each of the tumors occurring in the same kidney had unique somatic mutations, contributing to their different morphologies. So far, genomic characterization of multifocal renal tumors in TSC patients have not well been described. Tyburczy et al. reported that two patients with germline *TSC* mutation possessed distinct pathological features of multiple TSC-associated papillary RCCs and different second-hit mutations in *TSC1* or *TSC2* in the same patient, which may develop multifocal renal tumors. Interestingly, 35.2% of the somatic mutations identified in our papillary RCC sample were absent in typical papillary RCC, which might have led to the occurrence of TSC-associated papillary RCC in our patient. Moreover, driver mutations such as *PABPC1* and *DICER1* other than *TSC*1 or *TSC*2 may affect the TSC-associated tumor formation (**Figure 4**). Considering that 10%–15% of patients with TSC have no mutation in *TSC1* or *TSC2* as in our case, the acquisition of somatic mutations may also lead to the occurrence of multiple renal tumors with distinct phenotypes in these patients. These findings may contribute to further understanding the various aspects of TSC-associated RCC, although more cases are needed to fully elucidate this phenomenon.

In conclusion, our case report indicates that immunohistochemistry analysis is an important tool to diagnose TSC-associated papillary RCC. Moreover, our findings demonstrate that the accumulation of somatic mutation profiles is important to further understand the occurrence of TSC-associated RCC.

## Data Availability Statement

The original contributions presented in the study are included in the article/**Supplementary Material**. Further inquiries can be directed to the corresponding author.

## Ethics Statement

The studies involving human participants were reviewed and approved by Institutional Review Board of Osaka University (approval number: 668-5). The patients/participants provided their written informed consent to participate in this study.

## Author Contributions

TY performed data analysis and drafted the article. TK planned the entire project, performed data analysis, and completed the article. MU planned, supervised the entire project and completed the article. NN provided the study design and the working hypothesis and completed the article. KK conducted experiments, performed data analysis, and completed the article. MK and EM conducted experiments and completed the article. KH, AK, TU, SF, HK, RI, NI, and KF conducted data analysis and provided scientific advice. All authors contributed to the article and approved the submitted version.

## Funding

This work was supported by JSPS KAKENHI (Grant-in-Aid for Scientific Research (C), grant number 18K09133).

## Conflict of Interest

The authors declare that the research was conducted in the absence of any commercial or financial relationships that could be construed as a potential conflict of interest.

## Abbreviations

AMACR, α-methylacyl-CoA racemase; AML, angiomyolipoma; CT, Computed tomography; FFPE, Formalin-fixed paraffin-embedded; RCC, Renal cell carcinoma; SDHB, Succinate dehydrogenase subunit B; TSC, Tuberous sclerosis complex.
