# Supplementary material for: Genomic and Pathological Characterization of Multiple Renal Cell Carcinoma Regions in Patient With Tuberous Sclerosis Complex: A Case Report
Source: Front Oncol. 2021 May 12;11:691996. doi: 10.3389/fonc.2021.691996 (PMC8149899; doi:10.3389/fonc.2021.691996)
Supplement: Supplementary file 1 [file DataSheet_1.docx]

**Additional Materials for**

**Genomic and pathological characterization of multiple renal cell carcinoma regions in patient with tuberous sclerosis complex: a case report**

**Methods**

**Additional Figure 1**

**Methods**

***Immunohistochemical analysis***

Formalin-fixed paraffin-embedded (FFPE) tissues were cut into 5-μm-thick sections and the tissue slides were stained on a Ventana Benchmark Ultra System (Roche, Basel, Switzerland). Epitope retrieval solution I (Roche) was used for the 20-minute treatment. Anti-human CK7 (clone SP52, Roche), AMACR (clone 13H4, Dako, Agilent, Santa Clara, CA), CD10 (clone 56C6, Novocastra, Wetzlar, Germany), and SDHB (21A11AE7, Abcam, Cambridge, UK) antibodies were applied to the tissue sections. The antigen-antibody binding was visualized using a BOND polymer refine detection solution (Roche) according to the manufacturer’s instructions.

***Whole-exome sequencing and data analysis***

Genomic DNA was extracted from the FFPE tissues of the three tumors using the QIAamp DNA FFPE Tissue Kit (Qiagen, Valencia, CA). As germline control DNA, genomic DNA was extracted from peripheral blood mononuclear cells using an RNA mini kit (Qiagen). Whole-exome libraries were built using the SureSelectXT Human All Exon V5 kit (Agilent, Santa Clara, CA) and sequenced by 100‐bp paired-end reads on a HiSeq 2500 Sequencer (Illumina, San Diego, CA). The obtained sequence data were analyzed using an in-house pipeline as described previously([1](#_ENREF_1), [2](#_ENREF_2)).

**
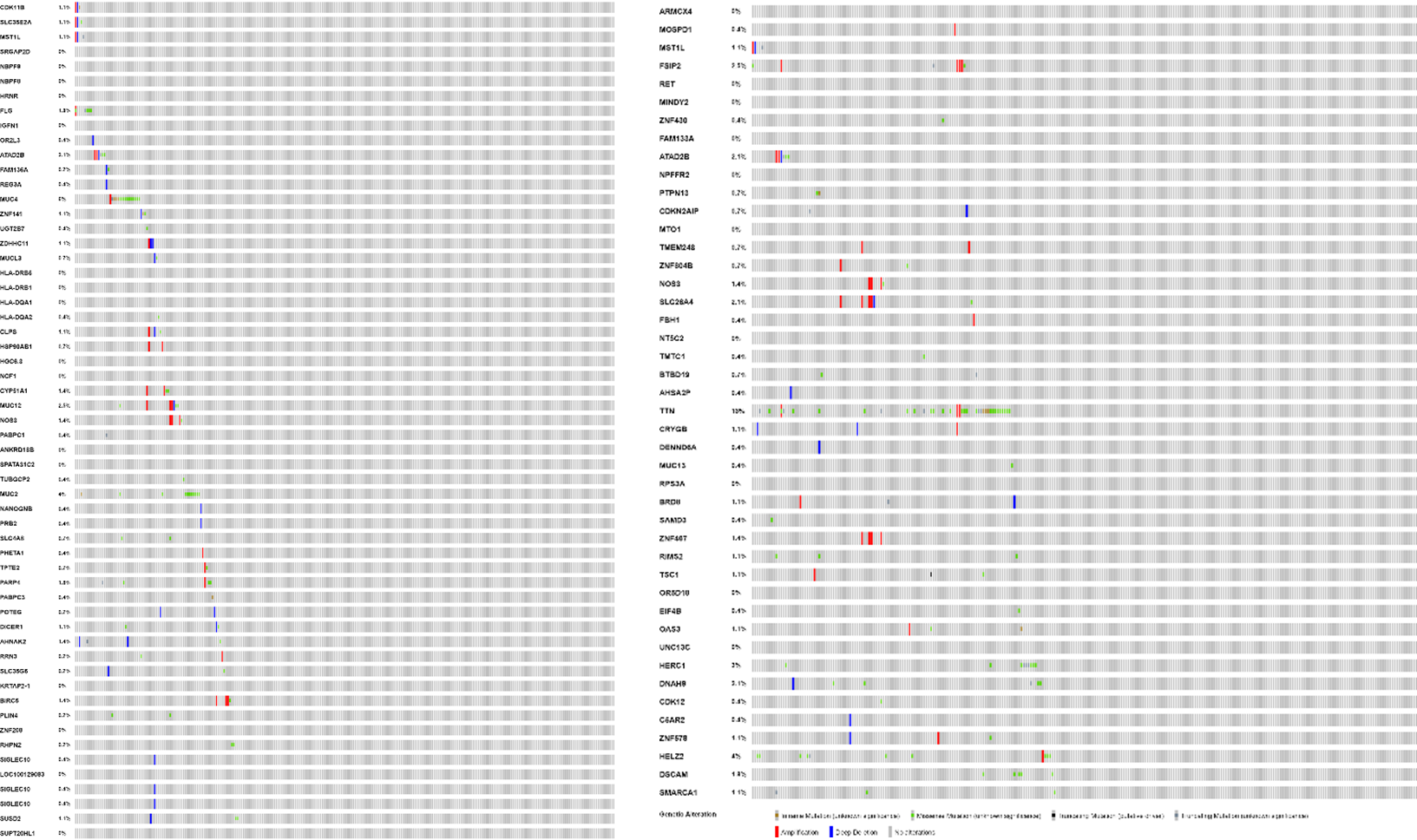
**

**Additional Figure 1. Mutational information in the papillary RCC**

Among 128 mutations in the papillary RCC in this patient, 45 mutations (35.2 %) were not found in TCGA database.

**References**

1. Kato T, Park JH, Kiyotani K, Ikeda Y, Miyoshi Y, Nakamura Y. Integrated analysis of somatic mutations and immune microenvironment of multiple regions in breast cancers. *Oncotarget*. (2017) 8:62029-38. doi: 10.18632/oncotarget.18790

2. Matsuda T, Leisegang M, Park JH, Ren L, Kato T, Ikeda Y, et al. Induction of Neoantigen-Specific Cytotoxic T Cells and Construction of T-cell Receptor-Engineered T Cells for Ovarian Cancer. *Clin Cancer Res*. (2018) 24:5357-67. doi: 10.1158/1078-0432.CCR-18-0142
